# P-1441. Inadequate Responses to Influenza Vaccination in Adolescents with Inflammatory Bowel Disease (IBD): Making the Case for Improved Strategies

**DOI:** 10.1093/ofid/ofaf695.1628

**Published:** 2026-01-11

**Authors:** Cristina Tomatis Souverbielle, Zhaohui Xu, Sherri Surman, Dylan Brown, Sara Mertz, Nicole Skinner, Marie Wehenkel, Guy Brock, Asuncion Mejias, Katherine Bline, Jennifer L Dotson, Brendan Boyle, Octavio Ramilo

**Affiliations:** Nationwide Children’s Hospital, Worthington, Ohio; St. Jude Children's Research Hospital, Memphis, TN; St. Jude Children's Research Hospital, Memphis, TN; Abigail Wexner Research Institute at Nationwide Children’s Hospital, Columbus, Ohio; The Research Institute at Nationwide Children's Hospital, Columbus, Ohio; Abigail Wexner Research Institute at Nationwide Children’s Hospital and The Ohio State University, Columbus, Ohio; St. Jude Children's Research Hospital, Memphis, TN; The Ohio State University, Columbus, Ohio; St Jude Children's Research Hospital, Memphis, TN; Nationwide Children's Hospital, Columbus, Ohio; Arkansas Children’s Hospital, University of Arkansas for Medical Sciences, Little Rock, Arkansas; Nationwide Children’s Hospital, Worthington, Ohio; St Jude Children’s Research Hospital, Memphis, Tennessee

## Abstract

**Background:**

Patients with inflammatory bowel disease (IBD) have up to 3 times higher rates of influenza hospitalizations than healthy individuals. In adolescents with other immunocompromising conditions studies suggest that higher or repeated influenza vaccination (FluIV) doses induce better responses. In children with IBD annual FluIV is recommended, but how their baseline immune dysregulation and immunosuppressive treatments alter vaccine immunogenicity is poorly understood.

Our goal was to evaluate longitudinal serologic and cellular responses after FluIV in IBD adolescents (IBDa) compared to healthy controls (HC).

**Figure 1.**
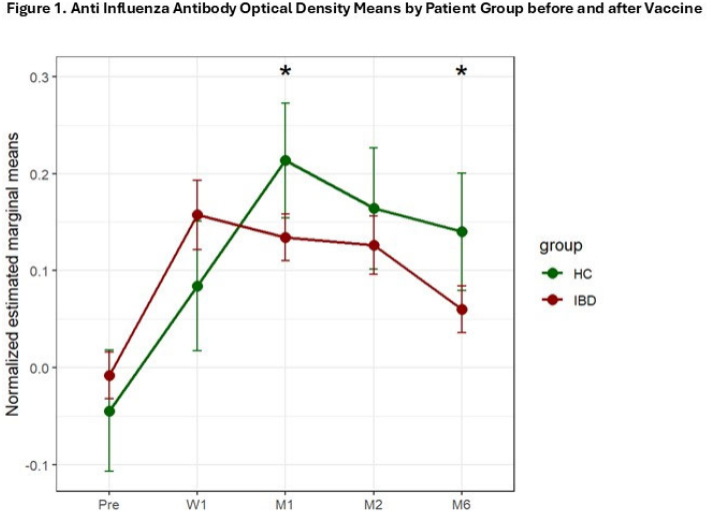
Anti-Influenza Antibody Optic Density Means by Patient Group before and after Vaccine

**Figure 2.**
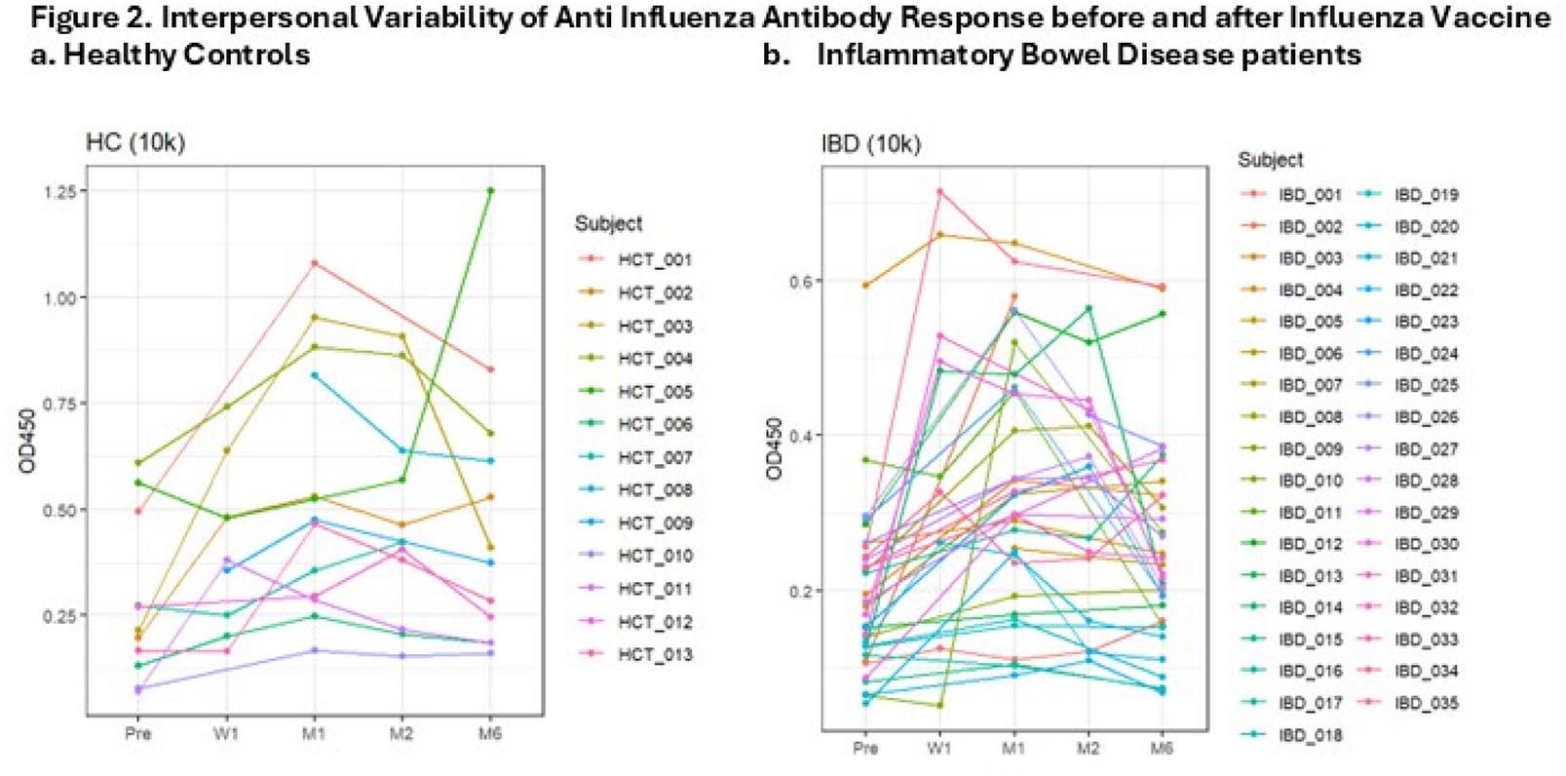
Interpersonal Variability of Anti-influenza Antibody Response before and after Vaccine

**Methods:**

IBDa (12-18 y) and matched HC vaccinated with the 2023-2024 inactivated quadrivalent FluIV were evaluated. Blood samples were obtained pre-vaccination (Pre-fluIV), 1 week (W1), 1 month (M1), 2 months (M2) and 6-8 months (M6) post-FluIV. Influenza antibodies (FluAb) were measured by ELISA and optical density (OD) values compared between groups using linear mixed models. In a subset of patients peripheral blood immunophenotype was analyzed, focusing on T-follicular helper cells (Tfh), T-regs, CD4 T-cells and B, which are key in humoral responses.

**Figure 3.**
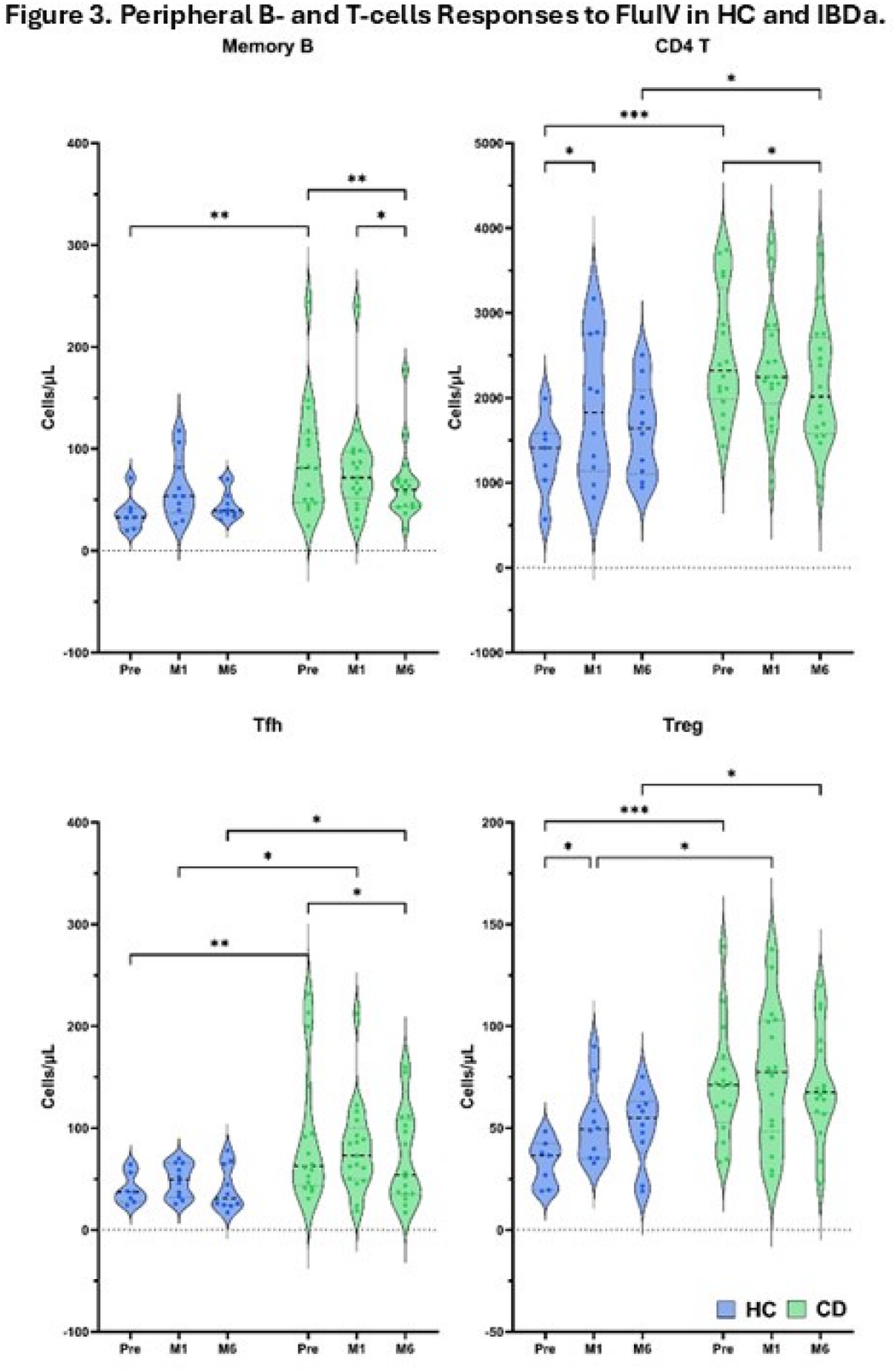
Peripheral B- and T-cells responses to FluIV in HC and IBDa.

**Results:**

From 9/2023-12/2023, we enrolled 35 IBDa, median age 16.5 (12-17) years, and 13 HC, median age 15.19 (12-16) years. 30% were female in both groups. Among IBDa 30 (86%) were treated with anti-TNF. Influenza specific antibodies were significantly lower in IBDa vs HC at 1 and 6 months (p=0.032, 0.036, Fig1), with significant variability within groups (Fig2). T and B cell phenotyping analyzed in 10 HC and 17 IBDa (all received anti-TNF), showed that IBDa had significantly higher CD4 T, Tfh, and Treg cells counts Pre-FluIV (p≤ 0.05), but decrease in Tfh cell counts from M1 to M6 (*P* = 0.055; Fig3). Further, memory B cells and CD4 T cells significantly decreased post vaccination only in IBDa (p< 0.05).

**Conclusion:**

Immune responses to FluIV differ between anti-TNF-treated IBDa and HC. Antibody responses to FluIV were significantly higher in HC vs IBDa and persisted until M6. A subset of IBDa had higher B and T cell counts Pre-FluIV, and limited B and T-cell responses post FluV, with significant individual variability in FluAb responses. These data suggest the need to improve FluIV strategies for IBDa.

**Disclosures:**

Cristina Tomatis Souverbielle, MD, Merck inc: Research support isp Asuncion Mejias, MD, PhD, MsCS, Enanta: Advisor/Consultant|Merck: Grant/Research Support|Moderna: Advisor/Consultant|Pfizer: Advisor/Consultant|Sanofi-Pasteur: Advisor/Consultant Brendan Boyle, MD, MPH, Endo-Therapeutics: Endoscopic device - royalites Octavio Ramilo, MD, Merck: Advisor/Consultant|Merck: Grant/Research Support|Merck: Honoraria|Moderna: Advisor/Consultant|Pfizer: Advisor/Consultant|Pfizer: Honoraria|Sanofi: Advisor/Consultant|Sanofi: Honoraria

